# Comparisons of myocardial strain and strain rate in patients with heart failure with preserved and reduced ejection fraction using Feature Tracking of cine MR images

**DOI:** 10.1186/1532-429X-16-S1-P300

**Published:** 2014-01-16

**Authors:** Gangadhara Kabbli, Lynette J Duncanson, Michael Passick, Joshua Y Cheng, Kathy Halloran, Jeannette McLaughlin, Jie J Cao

**Affiliations:** 1 Advance cardiac imaging, St. Francis Hospital., Roslyn, New York, USA

## Background

Cardiac magnetic resonance imaging (CMR) is an important modality in heart failure evaluation. The aim of this study was to compare myocardial mechanical properties between patients in heart failure with reduced ejection fraction (HFrEF) and patients in HF with preserved EF (HFpEF).

## Methods

All participants were prospectively recruited and underwent CMR in a 1.5 T scanner. LVEF < 45% and ≥45% were used to define HFrEF and HFpEF, respectively. Dilated cardiomyopathy was the primary cause for HFrEF. HF status was confirmed with B-type natriuretic peptide (BNP) > 400 pg/ml. Patients with myocardial infarction were excluded. Myocardial circumferential strain (CST) and strain rate (CSR) was analyzed in mid LV of the short axis plane and the longitudinal strain (LST) and strain rate (LSR) in 4-chamber view of the SSFP cine images using feature tracking (CIM software, Auckland, New Zealand). LV end diastolic pressure (LVEDP) was estimated using normalized left atrial transition time from time-intensity curves of the first pass perfusion images.

## Results

LVEF was 55 ± 2%, 58 ± 7% and 26 ± 9%, the median (range) BNP 15 (48) pg/ml, 443 (3250) pg/ml and 651 (2791) pg/ml, and the mean LVEDP 8 ± 2 mmHg 22 ± 13 mmHg and 22 ± 10 mmHg, in normal controls (N = 8), HFpEF (N = 11), and HFrEF (N = 13), respectively. The average CST in mid LV was -14.6 ± 3.4%, -13.8 ± 4.3% and -6.6 ± 3.6% (p < 0.001) while the LST was -14.1 ± 1.6%, -9.7 ± 5.2% and -6.5 ± 2.2%, respectively resulting in modest CST reduction (6%) but greater LST reduction (31%) in HFpEF, and similar CST (55%) and LST reduction (54%) in HFrEF using normal controls as reference. Similar magnitude of reduction was observed in CSR and LSR for both groups. As for the rate of relaxation in early diastole there was ~16% reduction in HFpEF and ~50% in HFrEF for both short and long axis analysis. In contrast, the rate of relaxation in late diastole was markedly increased (119% and 46%) in HFpEF and reduced (27% and 16%) in HFrEF.

## Conclusions

Feature tracking analysis of the CMR cine images demonstrated homogeneous strain and strain rate reduction in both the circumferential and longitudinal direction in HFrEF patients with dilated cardiomyopathy. In contrast, preserved circumferential strain of the mid LV coupled with reduced longitudinal strain and markedly increased rate of relaxation in late diastole are features of myocardial mechanical performance in HFpEF. Our findings suggest that feature tracking analysis of the cine images is promising in characterizing myocardial properties of patients with HF.

## Funding

None.

